# Addiction

**DOI:** 10.1192/j.eurpsy.2021.43

**Published:** 2021-08-13

**Authors:** J. Bramness

**Affiliations:** Department Of Alcohol, Drug And Tobacco Research, Norwegian Institute of Public Health, Oslo, Norway

## Abstract

**Addiction:**

what did we learn in 2020? Every year several thousand scientific papers on alcohol, drugs, and nicotine are published. The picking of five papers must obviously be arbitrary and subjective. However, the scientific literature of 2020 cannot be regarded without acknowledging the many papers concerning the COVID-19 pandemic. Some studies on alcohol, drug, and nicotine show a small increase, some a small decrease, but many no change. The addiction consequences of the pandemic and the societal lockdowns may thus be less dramatic than feared. This is true even if many papers reported higher mental distress during the pandemic and there is a close relationship between mental distress and substance use, a relationship that has been further confirmed in studies from the past year. Furthermore, a review concerning the addictive potential of cannabis has further alarmed us of the current liberalization also affecting Europe. A new figure of “1 in 3” cannabis users getting hooked may possibly replace the old “1 in 10”. Furthermore, the year has brought even more solid knowledge of the transition from substance-induced psychosis (SIP) to schizophrenia, teaching psychiatrists in acute psychiatry an important lesson on how to view SIP. As many as 1 in 3 patients with SIP will eventually receive a diagnosis of schizophrenia, making SIP the most powerful risk factor for schizophrenia known. Lastly, the lecture will present a very novel and unexpected finding regarding alcohol elimination, that may change how we treat intoxications with different alcohols.

**Disclosure:**

No significant relationships.

